# Machine Learning-Based Survival Prediction in Early-Stage Non-Small Cell Lung Cancer: Development and Cross-National External Validation

**DOI:** 10.3390/jcm15103701

**Published:** 2026-05-11

**Authors:** Nikhil Joshi, Hari Ponnamma Rani, Maxim Shevtsov, Thyageshwar Chandran

**Affiliations:** 1 Department of Mathematics, National Institute of Technology Warangal, Hanamkonda 506004, Telangana, India; nj24mac1r18@student.nitw.ac.in; 2 Department of Radiation Oncology, Klinikum Rechts der Isar, Technical University of Munich, 81675 Munich, Germany; 3 Department of Biotechnology, National Institute of Technology Warangal, Hanamkonda 506004, Telangana, India; thyagesh@nitw.ac.in

**Keywords:** lung cancer, non-small cell lung cancer, survival prediction, machine learning, random survival forest, external validation, SEER database

## Abstract

**Background:** Lung cancer remains one of the leading causes of cancer-related mortality worldwide. However, prognostic models developed within a specific population may not be accurate when applied to another population due to differences in demographics and clinical practices. In the present study, we investigated the cross-national applicability of machine learning (ML)-based survival prediction models trained on population data from the United States and validated on an independent Chinese clinical cohort. **Methods:** Cox proportional hazards, Random Survival Forest (RSF), and XGBoost-Cox models were developed and externally validated. Model discrimination was evaluated using the concordance index (C-index) and time-dependent AUC at 1, 3, and 5 years, along with calibration and decision curve analysis. Hyperparameter tuning was performed using cross-validation to reduce overfitting and improve model generalizability. **Results:** Three survival prediction models were developed using the U.S. SEER database (*n* = 13,260) and externally validated in an independent Chinese cohort (*n* = 505). Baseline characteristics differed between the cohorts, with the Chinese cohort being younger and having a higher proportion of stage IA disease. Despite these differences, all models demonstrated acceptable discrimination. The RSF model was the most stable across cohorts and time horizons, with a C-index of 0.740 (95% CI: 0.735–0.746) in SEER and 0.782 (95% CI: 0.720–0.844) in the Chinese cohort. RSF showed good calibration at 1 and 3 years but slightly overestimated 5-year mortality risk in the Chinese cohort. **Conclusions:** Machine learning-based survival prediction models, such as the Random Survival Forest model, are promising and robust tools for predicting cross-population survival in early-stage non-small cell lung cancer (NSCLC). However, differences in patient characteristics and treatment patterns may influence long-term model performance. These findings highlight the potential of flexible machine learning models in oncology and the essential role of rigorous external validation.

## 1. Introduction

Lung cancer remains a major global health burden and is one of the leading causes of cancer-related mortality worldwide, accounting for approximately 1.8 million deaths each year [1]. Although significant advances have been made in recent years in targeted therapy and immunotherapy, survival rates among patients with lung cancer still vary considerably. Outcomes are influenced by multiple factors, including patient age, cancer stage at diagnosis, histological subtype, and treatment modality, as well as comorbidities and access to care. Large population-based studies provide an opportunity to examine these determinants in real-world settings, offering insights that complement findings from clinical trials. The Surveillance, Epidemiology, and End Results (SEER) Program is a comprehensive, population-based database that captures cancer incidence, treatment, and survival data across diverse populations in the United States, making it a valuable resource for epidemiological and prognostic research [2]. It has been observed that prognostic models developed in a certain population might not be applicable to another population due to differences in ethnicity and healthcare systems [3]. This is particularly relevant when models are translated between Asian and Western populations, where differences in tumor biology, treatment practices, and patient demographics may substantially affect model performance [4].

Non-small cell lung cancer (NSCLC) accounts for approximately 85% of all lung cancer cases and represents a biologically heterogeneous group of tumors. It is characterized by diverse molecular alterations, including mutations in the epidermal growth factor receptor (EGFR) gene and rearrangements involving the anaplastic lymphoma kinase (ALK) gene. These genetic changes play a critical role in tumor initiation, progression, and metastasis, and they have a substantial impact on patient prognosis and survival outcomes. The identification of such driver mutations has transformed the clinical management of NSCLC by enabling the development of targeted therapies. Treatments directed against EGFR mutations and ALK rearrangements have demonstrated significant improvements in response rates and survival compared with conventional chemotherapy. As a result, molecular profiling has become an essential component of personalized treatment strategies for patients with NSCLC [5]. Interestingly, it has been well established that the frequency of these mutations varies across different ethnic and geographic populations. EGFR mutations are present in approximately 30–50% of lung adenocarcinoma cases among East Asian populations, compared with only about 10–15% among Western populations. In contrast, KRAS mutations are more prevalent in Western populations than in East Asian populations [6,7]. In addition, comprehensive genomic profiling of NSCLC has been conducted by The Cancer Genome Atlas (TCGA), which has further highlighted the molecular complexity and heterogeneity of the disease across patients [8]. These differences may influence disease progression and survival patterns across patient groups, reflecting variations in environmental exposures as well as the availability and selection of treatment options [9]. It is possible that predictive models developed using data from one population may not perform well in another, as model performance is known to deteriorate when applied to populations with different demographic and clinical characteristics [10]. This heterogeneity highlights the need to assess the cross-population applicability of machine learning-based survival prediction models in oncology [11].

The Cox proportional hazards model is the most widely used statistical method for time-to-event analysis in cancer studies [12], while the Kaplan–Meier estimator is commonly used to describe survival patterns without adjustment [13]. Although these methods are well established and easily interpretable, they rely on underlying assumptions such as proportional hazard rates and log-linear effects of covariates. In recent years, various machine learning methods have been proposed as flexible tools capable of capturing non-linear relationships and interactions within complex clinical datasets. These approaches are increasingly being applied to cancer diagnosis, prognosis, and treatment decision-making. Recent studies have demonstrated their utility in predicting recurrence and survival in early-stage NSCLC [14,15]. In particular, Random Survival Forests extend tree-based ensemble methods to right-censored survival data without requiring the proportional hazards assumption [16]. Similarly, gradient boosting algorithms such as XGBoost have been adapted for survival analysis by optimizing the Cox partial likelihood function [17]. However, an important question remains as to whether the flexibility of these algorithms translates into improved performance across diverse clinical populations.

Comparative studies in oncology have shown that machine learning algorithms can improve discrimination in survival analysis, typically measured using the concordance index [18] or time-dependent AUC [19]. However, many studies rely exclusively on internal validation methods, which may introduce bias in performance estimates, particularly when data distributions are relatively stable. From a methodological perspective, it is essential to validate machine learning models on independent populations to assess their transportability and calibration robustness [20]. Furthermore, the TRIPOD initiative emphasizes the importance of transparent development and validation of prognostic models [21]. Despite this, studies evaluating the transportability of machine learning models across U.S. population-based cancer registries and independent Chinese clinical cohorts remain limited.

In the present study, this research gap is addressed by developing three survival prediction models (i.e., Cox proportional hazards, Random Survival Forest (RSF), and Extreme Gradient Boosting with Cox loss (XGBoost-Cox)) using a large NSCLC patient cohort from the SEER database. Model performance is evaluated in terms of discrimination and calibration at 1, 3, and 5 years. Importantly, the models are externally validated on an independent Chinese patient cohort without further parameter tuning. By assessing predictive performance in a geographically and clinically distinct population, this study aims to evaluate model generalizability rather than merely optimizing internal performance. The data sources and preprocessing strategies are described in Section 2, the modeling approach in Section 3, and the clinical and methodological implications in Section 4. This study contributes to the literature by examining the generalizability of survival prediction models for early-stage NSCLC using SEER data and validating them in an independent Chinese cohort.

## 2. Materials and Methods

### 2.1. Source Data and Study Population

In this study, two publicly available datasets were used to develop and validate survival prediction models for patients with NSCLC. Patients diagnosed with early-stage NSCLC were included according to SEER staging criteria for stage I–II disease. Patients with missing survival status and/or incomplete clinicopathological information were excluded.

The primary dataset was obtained from the Surveillance, Epidemiology, and End Results (SEER) Program of the U.S. National Cancer Institute. The data were extracted from the SEER Research Data: 8 Registries, submitted in November 2023. This dataset includes cases diagnosed between 1975 and 2021, along with associated county-level information. Patients diagnosed with early-stage NSCLC during this period were selected based on SEER coding criteria. To externally validate the proposed survival prediction models in a non-U.S. population, a Chinese dataset was also used in this study. This dataset has previously been used for the development and validation of traditional prognostic nomograms for early-stage NSCLC [22]. In the present work, we extend this foundational analysis to evaluate the performance of advanced machine learning algorithms for NSCLC survival prediction in a broader, global context.

### 2.2. Variable Selection and Data Preprocessing

The variables required for analysis were extracted, including age at diagnosis, sex, tumor laterality, primary tumor site, tumor stage, N stage, T stage, tumor size, number of examined lymph nodes, number of positive lymph nodes, histological subtype, tumor grade, and treatment-related variables such as type of surgery and chemotherapy status. The outcome variable of interest was overall survival.

Prior to model development, all variables underwent preprocessing to ensure data quality and consistency. Continuous variables were categorized based on clinically relevant thresholds. Age was grouped into four categories: <50, 50–59, 60–69, and ≥70 years. Tumor size was similarly categorized into four groups: ≤9 mm, 10–19 mm, 20–29 mm, and ≥30 mm. Categorical clinical variables, including sex, tumor laterality, pathological stage, T stage, N stage, surgical procedure, chemotherapy status, primary tumor site, histological subtype, and tumor grade, were encoded as categorical factors with predefined reference levels to ensure consistent interpretation of model coefficients and predictions.

Missing data handling was performed prior to model training. The final analytic sample of 13,260 SEER patients was derived from an initial cohort of 51,125 patients through a combination of study design criteria and data quality exclusions. The primary reduction resulted from restricting the cohort to early-stage disease (stages IA–IIB) to ensure comparability with the Chinese validation cohort, along with corresponding restrictions on T classification (T1–T3) and N classification (N0–N1). Additional exclusions included patients with unknown or invalid tumor size measurements (*n* = 10,626), invalid lymph node examination codes (SEER codes 95, 98, and 99; *n* = 7777), undefined surgical procedure codes, and laterality values other than left or right.

Among the final analytic cohort, tumor grade was the only variable with a substantial proportion of missing values, with 3229 patients (24.4%) in the SEER cohort and 4 patients (0.8%) in the Chinese cohort recorded as unknown. All other covariates, including age, sex, laterality, primary site, pathological stage, T and N classification, tumor size, lymph node counts, surgery type, chemotherapy status, and histological subtype, had complete data in both cohorts after exclusions.

For tumor grade, missing values were retained as a distinct encoded category (code = 0), while observed grades were coded as Grade I = 1, Grade II = 2, Grade III = 3, and Grade IV = 4. In the Random Survival Forest and XGBoost-Cox models, this “unknown” category was treated as an independent level, allowing the algorithms to learn its potential association with survival outcomes during tree construction. Nodal status was encoded as a binary variable, where 0 indicated no confirmed lymph node positivity or unknown status, and 1 indicated confirmed positivity in one or more nodes. This strategy was chosen over complete-case analysis to preserve the full sample size and to avoid assuming that missingness in grade was completely at random, which cannot be verified in population-based registry data. The same encoding scheme was applied consistently across both cohorts to ensure harmonized preprocessing during external validation.

To assess robustness, a sensitivity analysis was conducted using the subset of 10,031 SEER patients (75.6%) with known tumor grade. The RSF and XGBoost-Cox models were refitted on this complete-case dataset. The resulting concordance index values were nearly identical to those of the primary analysis, with the RSF model yielding 0.739 versus 0.740 in the main analysis (difference: 0.001), and the XGBoost-Cox model showing similarly stable performance (0.732 vs. 0.730). These findings indicate that the missing-as-category approach did not introduce meaningful bias in model performance.

### 2.3. Statistical Analysis

Data collected during the study was subjected to statistical analysis using Python (version 3.12.12) software package. The baseline patient characteristics were analysed using descriptive statistics. The categorical variables were stated as number (*n*) and percentage (%) overall. The duration of survival of the patient was noted in months from the first diagnosis up until death due to any cause (or last follow-up). Initially, regression analysis with univariate and multivariate Cox proportional hazards were conducted in the SEER cohort to identify predictors of survival. Clinical factors were included as categorical based on clinically relevant groupings. Hazard ratios with their corresponding 95% confidence intervals were used to assess the association between predictors and patient survival. To determine the generalizability of the Cox regression model, the fitted Cox regression model of the SEER cohort was applied to the Chinese cohort without the re-estimation of the model parameters. This was done to compare model performance across patient populations with differing demographic characteristics and clinical settings.

### 2.4. Model Construction and Evaluation

Three modelling approaches were developed to predict survival outcomes: a traditional Cox proportional hazards model, a Random Survival Forest (RSF) model, and an Extreme Gradient Boosting with Cox regression (XGB-Cox) model. The Cox model, developed using the SEER dataset, served as the baseline statistical approach for comparison across all three models. The RSF model was implemented to better capture non-linear relationships and complex interactions between predictor variables without assuming proportional hazards. Hyperparameters were optimized using a grid search strategy combined with 10-fold cross-validation to ensure robust model selection and minimize overfitting. The optimal hyperparameters were: n_estimators = 300, max_features = “sqrt”, min_samples_split = 20, and min_samples_leaf = 10. Parallel computation was enabled (n_jobs = −1), and a fixed random seed (random_state = 42) was used to ensure reproducibility. The XGB-Cox model was developed to improve predictive performance by combining gradient boosting with the Cox partial likelihood framework using the SEER dataset. To ensure equal comparisons, all models were built using identical clinicopathological characteristics. The three models were developed using the SEER cohort, and an external validation was performed using the Chinese cohort. For both cohorts, identical feature definitions and evaluation procedures were used throughout validation. For measuring the uncertainty associated with model discrimination, a bootstrap resampling method was applied. A total of 1000 bootstrap samples were created by resampling with replacement from each of the cohorts. For each bootstrap sample, a new calculation of the concordance index (C-index) was performed by re-estimating the predicted risk scores from the original models. The distribution of bootstrap results can be used to calculate a 95% confidence interval by determining the 2.5th and 97.5th percentiles.

The discriminative power of the models was assessed by computing the concordance index, C-index. To assess the predictive validity of each of the models, the area under the ROC curve, denoted by AUC, was computed using 1-year, 3-year, and 5-year time periods. Finally, the predicted survival rates were compared to the actual survival rates during the corresponding time period to check if the average of the predicted probabilities by each of the models is closely aligned to the actual values. By applying these models to an independent Chinese cohort, this study assessed their robustness and transportability across populations with differing demographic and clinical characteristics, thereby evaluating their generalizability in a real-world setting.

## 3. Results

### 3.1. Baseline Characteristics

The study utilized two cohorts of patients with early-stage non-small cell lung cancer (NSCLC). The SEER cohort was larger, comprising 13,260 patients, while the Chinese cohort included 505 patients. Although both cohorts fall within the same disease category, they are not directly comparable due to important demographic and clinical differences.

One of the main differences between the cohorts is patient age. The Chinese cohort was notably younger, with approximately 49.1% of patients aged 55 years or below. In contrast, only 18.8% of patients in the SEER cohort were aged 59 years or below. Conversely, older patients (>70 years) were more prevalent in the SEER cohort (40.2%) compared with the Chinese cohort (18.0%).

Sex distribution also differed between cohorts, with a higher proportion of male patients in the Chinese cohort (64.8%) compared with the SEER cohort (53.7%).

Stage distribution showed a more pronounced divergence. In the Chinese cohort, the majority of patients were diagnosed at stage IA (88.1%), whereas this proportion was lower in the SEER cohort (52.9%). Accordingly, more advanced stages (IIA and IIB) were more frequently observed in the SEER dataset. Similarly, the Chinese cohort included a higher proportion of T1 and N0 disease, indicating generally earlier-stage presentation.

Differences were also observed in treatment patterns. Surgical procedures varied between the two cohorts, and chemotherapy was used less frequently in the Chinese dataset (8.1%) compared with the SEER dataset (19.7%). Additional differences were noted in tumor grade distribution and histological subtypes.

Overall, the two cohorts originate from distinct clinical and demographic settings. While this limits direct comparability, it provides a valuable opportunity to evaluate model performance under heterogeneous conditions. If a model performs well across such diverse populations, it may indicate stronger generalizability and robustness. These differences likely reflect variations in healthcare systems, screening practices, and underlying population characteristics. Detailed cohort characteristics are presented in Table 1.

### 3.2. Cox Analysis

A multivariable Cox proportional hazards model was fitted to the SEER cohort to identify independent predictors of overall survival. Among the 13,260 patients, there were 7367 observed events during follow-up. The model exhibited strong global significance (likelihood ratio χ^2^ = 4027.97; 34 degrees of freedom; *p* < 0.005) and provided excellent discrimination with a concordance index of 0.72 (Table 2).

Patients diagnosed at older ages had a higher risk of cancer-related mortality than those diagnosed at younger ages, with the risk increasing progressively with age at diagnosis. For men, the hazard ratios were significantly higher than those for women, and this difference became more pronounced with increasing age at diagnosis (*p* < 0.005 for all comparisons). Among the patients studied, male sex was independently correlated with poorer survival (HR = 1.29; 95% CI, 1.23–1.35). Tumor stage was consistently associated with the likelihood of dying from the disease even after an adjustment for covariates. Patients diagnosed with stage IA disease had a lower risk of death compared with those diagnosed with stage IB, IIA, or IIB disease (HR = 1.38, 1.51, and 1.63, respectively; *p* < 0.005). Larger tumor size indicates increased tumor burden and a higher likelihood of disease-related mortality. A greater number of lymph nodes examined was associated with a reduced risk of mortality, suggesting that examining more lymph nodes may enhance the accuracy of disease staging and reflect improved surgical quality. There was strong evidence of an association between the type of surgical treatment and survival, with certain procedures linked to greater risk reduction and others to increased risk, likely reflecting differences in underlying disease severity and patient condition influencing treatment selection. Administration of chemotherapy resulted in a statistically significant increase in overall survival (HR = 0.87; 95% CI, 0.82–0.93). Examination of a higher number of lymph nodes may improve staging accuracy and facilitate the detection and removal of micrometastatic disease, which in turn may contribute to improved survival outcomes. Patients with poorly differentiated or undifferentiated tumors had a higher risk of mortality compared to those with well-differentiated tumors. Overall, the evidence indicates that patient demographics, tumor characteristics (including size and grade), and treatment modalities each independently influence life expectancy in patients diagnosed with early-stage NSCLC. As a statistically validated baseline, the Cox model provides a foundational reference for subsequent machine learning–based survival prediction models and their external validation in the Chinese cohort.

### 3.3. Model Comparison and Final Model Identification

The predictive accuracy of the Cox proportional hazards model, RSF, and XGBoost-Cox were formally assessed using the C-index (Table 3) and time-dependent AUC at 1, 3, and 5 years in both the SEER development cohort and the external Chinese validation cohort (Table 4).

**Figure 1:**
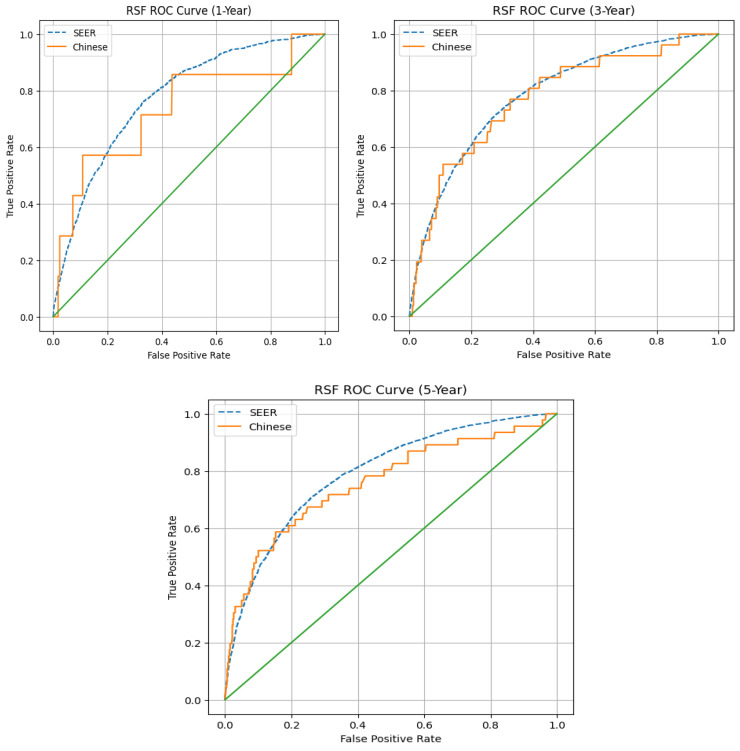
Time-dependent ROC curves at 1, 3, and 5 years. The green diagonal line represents the line of no discrimination corresponding to random prediction (AUC = 0.5).

The C-index for the Cox model in the SEER cohort was 0.717 (95% CI: 0.711–0.722) indicating that it was an acceptable discriminative tool. The machine learning models used in the current study had similar or slight improvements compared with the Cox model with respect to discrimination. The RSF model produced time-dependent AUC of 0.784, 0.794 and 0.800 at 1, 3 and 5 years, respectively. The XGB-Cox model produced time-dependent AUC of 0.787, 0.790 and 0.792 at 1, 3 and 5 years, respectively. For Chinese patients who were part of this study, the RSF produced AUC of 0.799, 0.790 and 0.749 at 1, 3 and 5 years, respectively. The XGB-Cox model produced AUC of 0.745, 0.757 and 0.744 these same time points. This is depicted in Figure 1.

Although RSF and XGB-Cox demonstrated similar overall discriminative ability, RSF exhibited relatively stable performance across time horizons and cohorts. In addition, RSF does not rely on proportional hazards assumptions and is capable of modeling nonlinear relationships and higher-order interactions among predictors. The model also provides variable importance measures that enhance interpretability and demonstrated satisfactory calibration at clinically relevant time points. Moreover, the machine learning models such as RSF are capable of capturing nonlinear relationship and complex interactions among the clinical variables. This could contribute to the stable predictive performance across the different populations. Based on its stable discrimination, flexibility in modeling complex predictor effects, and favorable calibration performance in both development and validation cohorts, the RSF model selected as the final predictive model for subsequent analyses, including receiver operating characteristic curve visualization, calibration assessment, and clinical interpretation.

**Figure 2:**
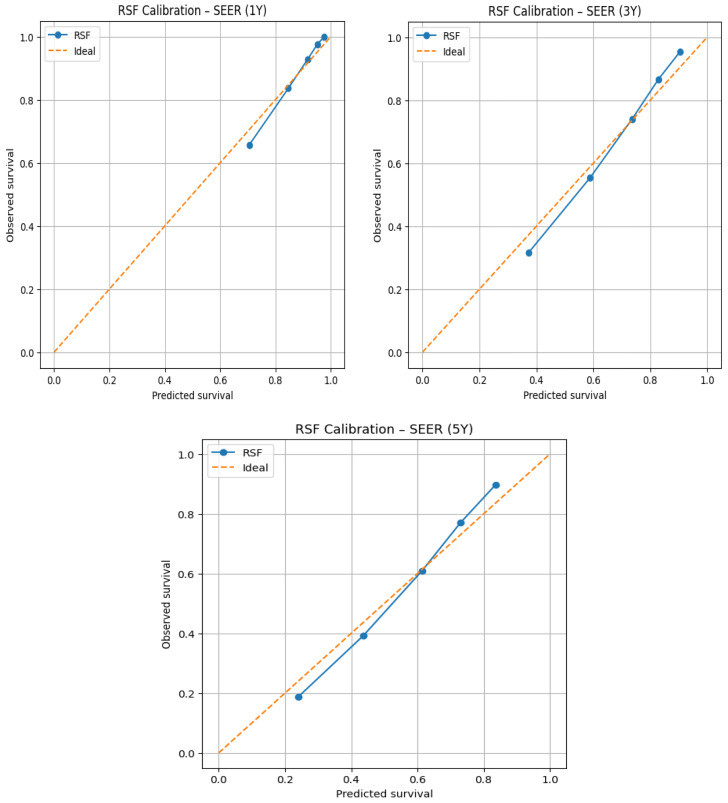
Calibration Plots for the RSF model in the SEER cohort at 1, 3, and 5 years.

**Figure 3:**
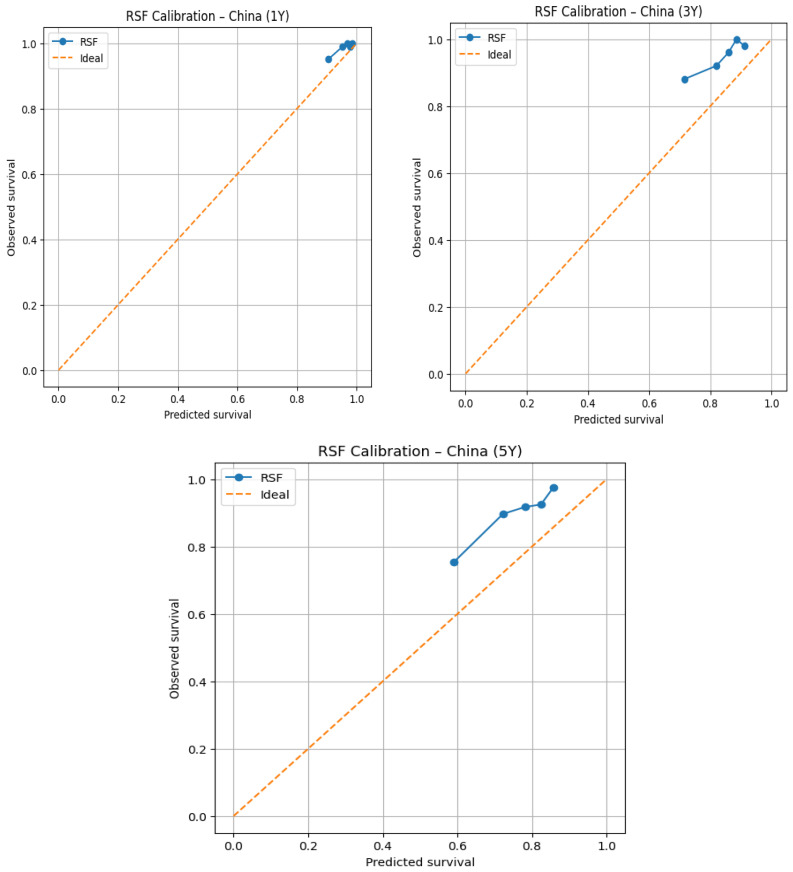
Calibration Plots for the RSF model in the Chinese cohort at 1, 3, and 5 years.

Figure 2 and Figure 3 display calibration plots demonstrating how accurately predicted probabilities of survival (as predicted by the RSF model) match with actual observed event probabilities of survival at 1, 3, and 5 years after diagnosis using data from the SEER development cohort and an external Chinese validation cohort.

Calibration plots include a dashed diagonal line (indicating perfect calibration) and compare probabilities predicted by the RSF model for survival with actual probabilities of survival for patients with matching risk levels (as defined by pre-established risk strata) to provide a means of visually assessing calibration. The RSF model appears to demonstrate good calibration at the 1st year for the external Chinese cohort, demonstrating that predicted survival probabilities from RSF align closely to actual observed outcomes among all 3 risk categories. This provides confirmation of the short-term survival prediction accuracy of the RSF model in the external cohort. At the 3-year time point, a mild divergence is observed between predicted and observed survival for the cohort of individuals with intermediate to high predicted survival probabilities. Specifically, the calibration curve appears to have shifted up relative to the perfect calibration line. This suggests that the RSF model is more likely to provide an overestimate mortality risk at this time point. At 5 years, a more marked divergence is observed between predicted and observed survival. The RSF model continues to overestimate long-term survival at this time point—a definitive indication of a decrease in calibration accuracy compared with the population that was used to develop the RSF model. Reasons for this divergence may include differences in the way the variables are distributed within the development versus external cohort populations, the continued change in treatment practices that occur, and the limited amount of longitudinal outcomes data available for the external cohort.

The development cohort of the SEER demonstrated high internal validity/accuracy at both early (1 year) and medium (3 year) follow-up time points based on the calibration of the RSF model across all risk stratification groups. Calibration remained strong at 3 years despite some minor differences across lower probability of survival groups indicating stable medium-term prediction performance. While the 5-year point-in-time calibration was less accurate than the previous time points, particularly in higher-risk patients with modest over predicted survival and calibration remained acceptable within the development cohort.

To formally quantify the calibration performance observed in the Chinese cohort, Expected/Observed (E/O) survival ratios and Brier scores were calculated at 1, 3, and 5 years, and logistic recalibration was subsequently applied. At 1 year, the RSF model demonstrated calibration with a mean predicted survival of 0.964 against an observed Kaplan–Meier survival of 0.986, yielding an E/O ratio of 0.978 and a Brier score of 0.014. Calibration deteriorated progressively at longer time horizons, with an E/O ratio of 0.900 and Brier score of 0.056 at 3 years (predicted 0.854 vs. observed 0.949), and an E/O ratio of 0.861 and Brier score of 0.111 at 5 years (predicted 0.771 vs. observed 0.895), statistically confirming the progressive overestimation of mortality risk observed in the calibration plots.

To further quantify this discrepancy, logistic recalibration was applied to the 5-year RSF predictions in the Chinese cohort. Prior to recalibration, the model predicted a mean 5-year event probability of 0.229 against an observed mortality rate of 0.091, yielding an E/O ratio of 2.519, confirming overestimation of mortality risk by approximately 2.5-fold. The recalibration intercept of −0.731 statistically confirmed systematic overestimation of overall event risk, while the recalibration slope of 1.460 indicated compression of the predicted risk distribution relative to the true risk distribution in the Chinese population. Following recalibration, the mean predicted event probability was corrected to 0.091, exactly matching the observed mortality rate, with the E/O ratio improving from 2.519 to 1.000, demonstrating that simple post hoc recalibration fully corrects the systematic bias introduced by cross-population differences in baseline mortality.

In conclusion, the RSF model exhibited good calibration at short term time horizons in both patient cohorts. Long-term (5 years) calibration demonstrated greater variability (observed in the external Chinese cohort) which may be impacted by covariate shift and the differences in follow-up duration. Therefore, these results further emphasize the value of external validation prior to using prognostic models outside of the population in which they were derived.

### 3.4. Decision Curve Analysis

**Figure 4:**
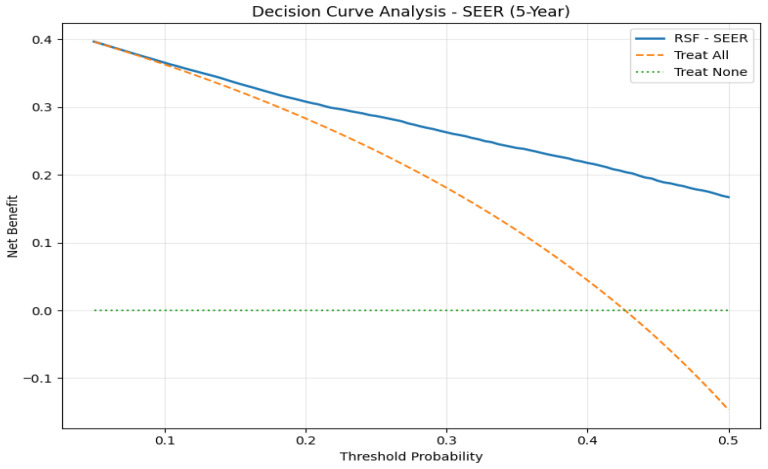
Decision curve analysis for 5-year overall survival in the SEER cohort. The RSF model demonstrates superior net benefit compared with treat-all and treat-none strategies across a broad range of threshold probabilities.

**Figure 5:**
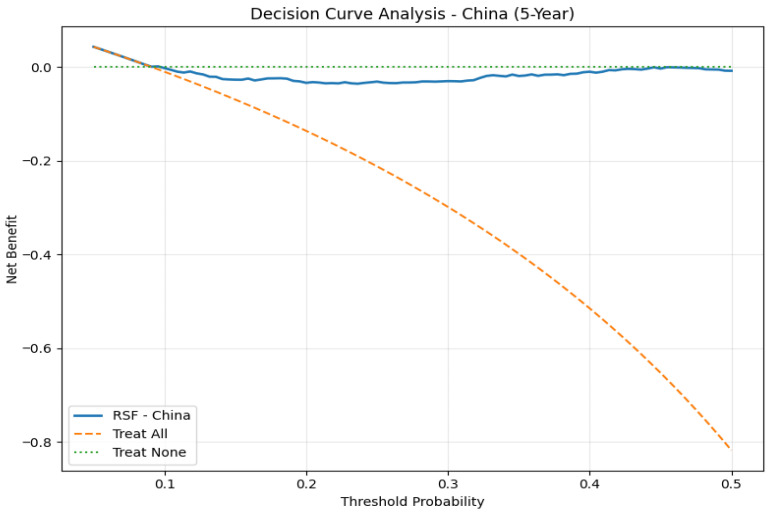
Decision curve analysis for 5-year overall survival in the external China cohort. The RSF model shows limited incremental net benefit compared with the treat-none strategy.

The 5-year survival percentage was calculated at 42.7% for the SEER dataset. DCA showed that the RSF model had a better net benefit than the ‘treat all’ and ‘treat none’ strategy for all values of threshold probability. The net benefit of the RSF model was found to be consistently above zero for all values of threshold probability. This implies the clinical utility of the RSF model for patients who are at high risk of disease recurrence and may benefit from enhanced surveillance and adjuvant treatments (Figure 4). The 5-year mortality percentage was found to be 9.1% for the China cohort dataset. The RSF model was found to have good discriminatory ability, although the net benefit was found to be low and similar to the ‘treat none’ strategy for all values of the threshold probability (Figure 5). This may be attributed to the low baseline percentage used for analysis. The RSF model may not have significant utility for populations with low baseline mortality. The study above indicates that the RSF model may have valuable clinical implications for the treatment of patients at an elevated risk of death. Through the monitoring and treatment of the concerned patients, this may help in their recovery. Furthermore, the importance of external validation and determination of clinical usefulness for regular clinical use in the treatment of oncological patients is highlighted in the present studies.

### 3.5. Risk Stratification Using RSF Model

**Figure 6:**
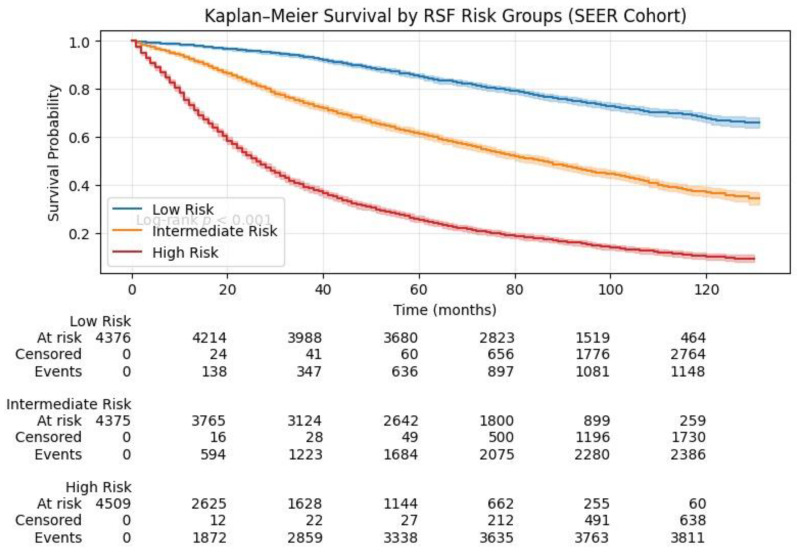
Kaplan–Meier survival curves for low-, intermediate-, and high-risk groups defined by the RSF model in the SEER cohort.

**Figure 7:**
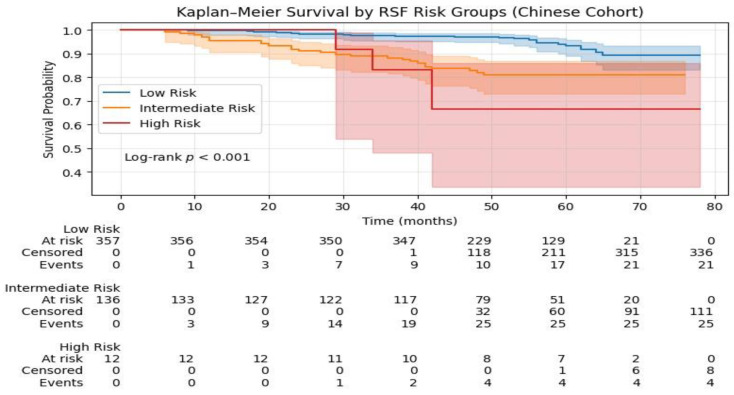
Kaplan–Meier survival curves for RSF-derived risk groups in the Chinese validation cohort.

To test the clinical applicability of the RSF model, the stratified risk groups were divided into three categories: low risk, intermediate risk, and high risk based on the predicted risk score of each patient by the RSF model. Cut-offs were set based on the tertiles of the predicted risk score from the SEER development dataset. Kaplan–Meier survival plots were constructed to compare the survival of each risk group (as shown in Figure 6 and Figure 7). In the SEER dataset, the survival plots were well separated, confirming the good discriminative power of the RSF model. Patients in the high-risk group had significantly worse survival compared to those in the intermediate and low-risk groups (log rank test, *p* < 0.001). Similar risk stratification was observed in the Chinese validation dataset, suggesting that the RSF model was applicable in different populations.

## 4. Discussion

In the present study, we have successfully developed and validated three survival prediction models for the patients with early-stage NSCLC. In addition, we have shown the predictive performance of the models. We have built these models for the patients with NSCLC using the data from the U.S. SEER registry. Then we have validated the performance of the models by using an independent Chinese cohort of patients with early-stage NSCLC. The survival prediction for the patients with NSCLC is still a challenge due to the differences among the patients and the treatment methods [23,24]. In the past few years, various studies have attempted to build prognostic models for the patients with NSCLC using the machine learning methods [25]. Also, considering biological heterogeneity, the model may present complex results based on differences in molecular characteristics and microenvironment, which may influence survival outcomes among different populations. Although all three models showed acceptable discriminatory ability, RSF showed the best consistency over time among the two distinct populations. The Cox PH model is an effective tool for survival analysis in oncology studies due to their transparency and ease of understanding [12]. In our study, the model was found effective for the SEER dataset (C-index 0.717) and had similar discriminatory power for the Chinese validation dataset, proving the worth of the model as an effective tool for baseline analysis. Cox assumes proportional hazards between predictors and outcomes and it also assumes a linear relationship between predictors and outcomes. These assumptions may not apply to how predictive models will reflect real-world clinical outcomes Effective methods such as RSF can easily detect non-linear relationships without any constraints [16,26]. Gradient boosting strategies including XGBoost-Cox, have shown promising results in recent oncologic prediction studies [27]. A major issue in developing prognostic models is the issue of generalizability. That is, does the performance of a given prognostic model generalize from the original dataset to another? In previous studies of prognostic models within the field of oncology, similar concerns have been raised regarding the tendency of predictive models to have diminished performance when validated in an independent external dataset [28]. Indeed, even when the same disease is examined, the populations studied may be quite disparate in terms of age, disease stage, tumor burden, and treatment paradigms [29].

In our analysis, the Chinese population consisted of younger patients with earlier disease stage, smaller tumor size, and surgical approach when compared to the SEER data. While the models continued to separate higher and lower-risk patients relatively well, we noted some changes in long-term calibration over five years. This indicates that although relative ordering in risk is well handled but the accuracy of absolute survival probabilities is less clear over long-term follow-up. These results support previous methodological studies in this area, which emphasized that transportability of models should not be based on discrimination results only, and that long-term calibration should be taken into consideration in external validation [19,30]. In the analysis of variable importance in the RSF model, it was noticed that the order of importance was as follows: the most important factor was the category of surgical treatment, followed by age, tumor size, sex, grade, and finally nodal evaluation. This is logical as surgery is the primary treatment modality for early-stage NSCLC [31]. On the other hand, the heterogeneity in the provision and practice of surgery may partially explain the variability in model performance between cohorts. Although ML models are often criticized for their lack of interpretability, RSF variable importance rankings provided an insightful understanding of which factors were driving the prediction process, which may help to dispel the idea that ML methods are used as “black boxes” [32]. With regard to the methodological implications, our research further reinforces the necessity for external validation in prognostic research. There is now a consensus that a model is able to perform well in the dataset in which it was trained, but it does not always do so in a different clinical group. These statements are further supported in TRIPOD framework guidelines.

In the current study, we made a decision to use the SEER-trained models on the Chinese cohort without any further fitting or adjustment. This method allowed us to analyze the performance of these systems under transport conditions. Hence, we are able to obtain a more accurate understanding of the strengths and weaknesses of these models in different populations. It would be most useful in survival models in terms of providing information in a timely manner, such as in counselling a patient, deciding on follow-up time, or deciding on treatment in early-stage NSCLC. In our research, we observed that the RSF showed good discrimination in both populations. This suggests that the RSF would be effective in differentiating between high and low risk in another population group. However, the drift in calibration at longer time points suggests that there should not be an assumption of probability in a new system of care. However, before it is applied clinically there is a need of recalibration especially if there are differences in treatment approaches from those in the development cohort. The variation in performance of the Chinese cohort indicates a major issue in transportability. Models trained on a specific population may not generalize well across different healthcare settings, and recalibration is likely necessary when substantial differences exist in case-mix, baseline event rates, or treatment practices between development and validation cohorts [33].

From a clinical perspective, the findings suggest that the predictive models may be integrated into routine practice to stratify patients into low-, intermediate-, and high-risk groups using readily available clinicopathological variables. This may support decision-making regarding follow-up intensity and adjuvant treatment. Beyond risk stratification, these findings also have implications for surgical decision-making in NSCLC. In selected patients with locally advanced disease involving structures such as the superior vena cava (SVC), en bloc resection with vascular reconstruction may be considered within a multimodality treatment strategy when complete resection is achievable [34,35]. For early-stage disease, minimally invasive approaches such as VATS or robotic surgery are preferred due to reduced morbidity, while conversion to thoracotomy may benefit from techniques such as modified pericostal suturing to reduce postoperative pain and preserve chest wall integrity [36]. Additionally, while lobectomy remains the standard surgical approach for early-stage NSCLC, segmentectomy may be appropriate in selected small peripheral tumors provided that adequate margins and systematic lymph node dissection are ensured [37,38]. These considerations highlight the importance of surgical quality, extent of resection, and patient selection in determining outcomes. Rare entities such as pulmonary inflammatory myofibroblastic tumors should also be considered in differential diagnosis, as they may mimic malignancy but often have distinct management pathways, including potential targeted therapy in ALK-positive cases [39]. Finally, the RSF-derived risk stratification may help clinicians tailor follow-up schedules and consider earlier intervention in high-risk patients, while avoiding overtreatment in low-risk groups, thereby improving resource allocation and patient care [40].

The limitations of the present study are as follows. Firstly, differences in baseline characteristics and treatment distributions between the cohorts make direct comparisons challenging. Secondly, detailed molecular biomarkers and information on systemic therapy were not available, which may influence model performance. Thirdly, the sample size and duration of follow-up in the Chinese cohort were smaller than those in the SEER cohort, which may affect the reliability of long-term calibration assessment. Lastly, although the RSF model provides variable importance measures, these are less intuitive than hazard ratios derived from Cox regression models.

In addition, key molecular biomarkers such as EGFR mutations, ALK rearrangements, and PD-L1 expression status were not available. Smoking history and environmental exposure factors may also influence model accuracy and were not included. Important clinical variables, including body mass index (BMI) and the American Society of Anesthesiologists (ASA) physical status score, were also unavailable in both the SEER database and the external validation cohort. These factors may affect patient fitness, perioperative risk, and survival outcomes, and their absence may introduce residual confounding. Overall, these limitations reflect the inherent constraints of population-based registry data and should be taken into account when interpreting the results.

## 5. Conclusions

In this cross-national validation study of early-stage NSCLC, we have systematically assessed the generalizability of survival prediction models developed in a large population-based study in the United States to an independent clinical study in China. Although there were substantial differences in the demographics, stage distribution, and general characteristics of the population being modeled in the two cohorts, the machine learning-based survival prediction models (Random Survival Forest Model) showed stable discriminatory ability in both populations. Additionally, the mere provision of discrimination interpretations is insufficient to fully describe cross-population model performance, and both calibration and clinical utility and the analysis provided additional important measures of performance.

Several limitations of the external validation cohort must be acknowledged. The Chinese cohort was relatively small (*n* = 505) with shorter follow-up duration, reducing statistical power for robust calibration assessment, particularly at the 5-year time horizon. The cohort also exhibited a markedly skewed stage distribution (88.1% Stage IA) and very low 5-year mortality (9.1%), which limits the ability to adequately test the model’s discrimination and clinical utility across the full spectrum of early-stage disease. Additionally, the fundamental difference between a population-based registry (SEER) and a single-institution clinical cohort (Chinese dataset) introduces inherent selection differences that cannot be fully adjusted for analytically. These findings should therefore be interpreted as preliminary evidence of cross-national transportability, pending validation in larger and more representative Asian cohorts with longer follow-up.

These results indicate that although there is flexibility in machine learning survival prediction models for generalizing across highly diverse populations, continuous model recalibration based on performed external validation remains critical for any clinical use of these models. In conclusion, this study provides support for the reasonable but promising use of machine learning-based survival prediction models in varying international oncology settings.

## Figures and Tables

**Table 1:** Baseline characteristics of the SEER and Chinese cohorts.

Variable	Category	SEER (N = 13,260)	Chinese (N = 505)
**Age group**	<50 years	451 (3.4%)	119 (23.6%)
	50–59 years	2042 (15.4%)	129 (25.5%)
	60–69 years	5436 (41.0%)	166 (32.9%)
	≥70 years	5331 (40.2%)	91 (18.0%)
**Sex**	Male	7124 (53.7%)	327 (64.8%)
	Female	6136 (46.3%)	178 (35.2%)
**Laterality**	Left	5608 (42.3%)	213 (42.2%)
	Right	7652 (57.7%)	292 (57.8%)
**Primary site**	Upper lobe (341)	7771 (58.6%)	308 (61.0%)
	Lower lobe (343)	4347 (32.8%)	44 (8.7%)
	Lung, NOS (349)	161 (1.2%)	140 (27.7%)
	Others (340, 342, 348)	981 (7.4%)	13 (2.6%)
**Stage**	Stage IA	7019 (52.9%)	445 (88.1%)
	Stage IB	3005 (22.7%)	42 (8.3%)
	Stage IIA	1625 (12.3%)	10 (2.0%)
	Stage IIB	1611 (12.1%)	8 (1.6%)
**Tumor stage (T)**	T1	7510 (56.6%)	416 (82.4%)
	T2	4338 (32.7%)	61 (12.1%)
	T3	1412 (10.6%)	28 (5.5%)
**Positive lymph nodes status**	N0	12,004 (90.5%)	490 (97.0%)
	N+	1256 (9.5%)	15 (3.0%)
**Tumor size**	<10 mm	799 (6.0%)	233 (46.1%)
	10–19 mm	4505 (34.0%)	182 (36.0%)
	20–29 mm	3560 (26.8%)	51 (10.1%)
	>29 mm	4396 (33.2%)	39 (7.7%)
**Lymph nodes examined**	0	4904 (37.0%)	97 (19.2%)
	1–9	4519 (34.1%)	206 (40.8%)
	10–19	2533 (19.1%)	140 (27.7%)
	20–29	682 (5.1%)	49 (9.7%)
	>29	622 (4.7%)	13 (2.6%)
**Chemotherapy**	No	10,652 (80.3%)	464 (91.9%)
	Yes	2608 (19.7%)	41 (8.1%)
**Grade**	Grade I	2239 (16.9%)	307 (60.8%)
	Grade II	4445 (33.5%)	97 (19.2%)
	Grade III	3133 (23.6%)	60 (11.9%)
	Grade IV	214 (1.6%)	37 (7.3%)
	Unknown	3229 (24.4%)	4 (0.8%)
**Surgery**	Sub-lobectomy	2079 (15.7%)	358 (70.9%)
	Lobectomy	6800 (51.3%)	145 (28.7%)
	Pneumonectomy	204 (1.5%)	1 (0.2%)
	Palliative/no surgery	4177 (31.5%)	1 (0.2%)
**Tumor type**	Adenocarcinoma	4878 (36.8%)	473 (93.7%)
	Squamous cell carcinoma	2826 (21.3%)	25 (5.0%)
	Adenosquamous carcinoma	178 (1.3%)	1 (0.2%)
	Large cell carcinoma	953 (7.2%)	3 (0.6%)
	Others	4425 (33.4%)	3 (0.6%)

**Table 2:** Multivariable Cox proportional hazards regression analysis of overall survival (*n* = 13,260).

Variable	HR (95% CI)	*p*-Value
**Age Group (Ref: <50 years)**		
50–59 years	1.54 (1.27–1.88)	*p* < 0.005
60–69 years	1.93 (1.60–2.33)	*p* < 0.005
≥70 years	2.46 (2.04–2.97)	*p* < 0.005
**Sex (Ref: Female)**		
Male	1.29 (1.23–1.35)	*p* < 0.005
**Laterality (Ref: Left)**		
Right	1.03 (0.99–1.08)	0.18
**Stage (Ref: Stage IA)**		
Stage IB	1.38 (1.18–1.62)	*p* < 0.005
Stage IIA	1.51 (1.26–1.82)	*p* < 0.005
Stage IIB	1.63 (1.20–2.20)	*p* < 0.005
**T Classification (Ref: T1)**		
T2	0.90 (0.77–1.06)	0.21
T3	0.99 (0.73–1.34)	0.93
**N Classification (Ref: N0)**		
N1	1.21 (1.02–1.44)	0.03
**Tumor Size (Ref: <10 mm)**		
10–19 mm	1.11 (0.99–1.24)	0.08
20–29 mm	1.28 (1.14–1.44)	*p* < 0.005
>29 mm	1.44 (1.27–1.64)	*p* < 0.005
**Lymph Nodes Examined (Ref: 0 nodes)**		
1–9 nodes	0.79 (0.72–0.87)	*p* < 0.005
10–19 nodes	0.72 (0.65–0.80)	*p* < 0.005
20–29 nodes	0.69 (0.60–0.80)	*p* < 0.005
>29 nodes	0.76 (0.65–0.87)	*p* < 0.005
**Positive Lymph Nodes (Ref: None)**		
Present	1.15 (0.99–1.33)	0.07
**Surgery (Ref: Sub-lobectomy)**		
Lobectomy	0.73 (0.67–0.79)	*p* < 0.005
Pneumonectomy	0.91 (0.74–1.11)	0.35
Palliative/no surgery	1.92 (1.75–2.11)	*p* < 0.005
**Chemotherapy (Ref: No)**		
Yes	0.87 (0.82–0.93)	*p* < 0.005
**Primary Tumor Site (Ref: Upper lobe)**		
Lower lobe	1.05 (0.99–1.10)	0.08
Lung, NOS	1.14 (0.94–1.38)	0.17
Others	0.93 (0.85–1.02)	0.14
**Tumour Type (Ref: Adenocarcinoma)**		
Squamous cell carcinoma	1.22 (1.15–1.29)	*p* < 0.005
Adenosquamous carcinoma	1.18 (0.98–1.43)	0.07
Large cell carcinoma	1.19 (1.09–1.30)	*p* < 0.005
Others	0.86 (0.81–0.91)	*p* < 0.005
**Tumor Grade (Ref: Unknown)**		
Grade I	0.71 (0.64–0.77)	*p* < 0.005
Grade II	1.02 (0.95–1.09)	0.67
Grade III	1.13 (1.06–1.21)	*p* < 0.005
Grade IV	1.20 (1.01–1.43)	0.04

**Model Performance:** Concordance index = 0.72. Partial AIC = 129,430.54. Likelihood ratio test = 4027.97 (*df* = 34), *p* < 0.005.

**Table 3:** Comparison of model performance in the SEER and Chinese cohorts.

Model	SEER C-Index (95% CI)	Chinese C-Index (95% CI)
Cox Proportional Hazards model	0.717 (0.711–0.722)	0.780 (0.717–0.843)
Random Survival Forest	0.740 (0.735–0.746)	0.782 (0.720–0.844)
XGBoost Cox model	0.732 (0.727–0.738)	0.726 (0.645–0.807)

**Table 4:** Time-dependent AUC comparison at 1, 3, and 5 years.

Cohort	Model	1-Year	3-Year	5-Year
SEER	Cox	0.774	0.775	0.774
	XGB-Cox	0.787	0.790	0.792
	RSF	0.784	0.794	0.800
Chinese	Cox	0.797	0.787	0.760
	XGB-Cox	0.745	0.757	0.744
	RSF	0.799	0.790	0.749

## Data Availability

The SEER data used in this study are publicly available from the Surveillance, Epidemiology, and End Results (SEER) Program of the U.S. National Cancer Institute (https://seer.cancer.gov/) accessed on 22 July 2025 Access requires completion of the SEER Data Use Agreement. The external validation dataset was obtained from the publicly available data of a previously published study [22].

## Abbreviations

The following abbreviations are used in this manuscript:

SEER	Surveillance, Epidemiology, and End Results
NSCLC	Non-Small Cell Lung Cancer
RSF	Random Survival Forest
XGB	Extreme Gradient Boosting
DCA	Decision Curve Analysis
EGFR	Epidermal Growth Factor Receptor
KRAS	Kirsten rat sarcoma viral oncogene homolog
ALK	Anaplastic Lymphoma Kinase
